# A rare adult case of primary uterine rhabdomyosarcoma with mixed pattern: a clinicopathological & immunohistochemical study with literature review

**DOI:** 10.1186/s13000-024-01518-w

**Published:** 2024-07-17

**Authors:** Nehal K.H. Kamel, Eiman Adel Hasby

**Affiliations:** Pathology Department, Faculty of Medicine, Tanta University, Tanta, Gharbia Egypt

**Keywords:** Immunohistochemistry, Olig-2, PAX3/7::FOXO1, Rhabdomyosarcoma, Uterus

## Abstract

**Background:**

Rhabdomyosarcomas are aggressive tumors that comprise a group of morphologically similar but biologically diverse lesions. Owing to its rarity, Mixed pattern RMS (ARMS and ERMS) constitutes a diagnostic and therapeutic dilemma.

**Case:**

Herein is presented a very rare case of mixed alveolar & embryonal rhabdomyosarcoma in the uterus of a 68-year-old woman. The wall of the uterine corpus & cervix was replaced by multiple whitish–yellow, firm nodules, measuring up to 12 cm. Microscopically, the tumor was predominantly composed of round to polygonal cells arranged in nests with alveolar pattern intermingled with hypo- & hypercellular areas of more primitive cells with scattered multinucleated giant cells seen as well. Extensive sampling failed to show epithelial elements. Immunohistochemical staining showed positive staining for vimentin, desmin, myogenin, CD56 & WT-1. However, no staining was detected for CK, LCA, CD10, ER, SMA, CD99, S100, Cyclin-D1 & Olig-2. Metastatic deposits were found in the peritoneum. The patient received postoperative chemotherapy and radiotherapy but died of systemic metastases 3 months after surgery.

**Conclusion:**

The rarity of this histological tumor entity and its aggressive behavior and poor prognosis grab attention to improving recognition and treatment modalities in adults.

## Introduction

Rhabdomyosarcoma (RMS) is an aggressive malignant mesenchymal tumor of striated muscle origin that is more commonly diagnosed in children and adolescents than adults [1]. It develops essentially in the deep soft tissue of the neck, extremities, and perineal region [2]. According to the World health organization (WHO) classification introduced in 2020, rhabdomyosarcoma is subclassified into four major subtypes: embryonal (ERMS), alveolar (ARMS), pleomorphic (PRMS), and spindle cell/sclerosing [3]. Primary uterine rhabdomyosarcoma can present as a heterologous differentiation in uterine carcinosarcoma or adenosarcoma or, far less commonly, arises as a pure uterine rhabdomyosarcoma [4, 5].

Primary pure rhabdomyosarcoma infrequently involves gynecological regions, where the embryonal subtype represents more than 75% of cases, especially in children with DICER1 syndrome, and is associated with favorable prognosis in comparison with ARMS and PRMS [3]. ARMS and PRMS are seen nearly exclusively in adults, with PRMS typically involving post-menopausal females [6]. Some rhabdomyosarcomas contain histologic features of multiple subtypes. In 1995, Pappo et al. reported that the presence of any alveolar element translates into a bad prognosis. [7]

The biologic basis for these mixed tumors is currently unknown, although some studies suggest that even the embryonal elements of “bad” tumors have genetic features of ARMS [8, 9].Rhabdomyosarcoma with mixed embryonal and alveolar features were previously thought to be a form of alveolar RMS, but studies have shown that most lack PAX3/7::FOXO1 fusions, suggesting that such tumors are more in line with embryonal RMS. However some mixed tumors have had detectable gene fusions which clearly would be more in keeping with alveolar RMS [10].

Owing to its rarity, there are limited data regarding frequency and clinico-pathological features of primary pure uterine rhabdomyosarcoma in publications. Therefore, the current study describes the clinicopathologic & immunohistochemical features of a new case of uterine RMS in an adult woman and also reviews the available cytological and clinicopathological findings of previously reported adult uterine RMS cases in English literature with the goal of improving recognition of this tumor outside of its classical setting.

**• Case:**

## Material and method

### Clinical data

Female patient aged 68 years presented with an abdominal mass and abnormal uterine bleeding. No specific medical or surgical history (including a history of previous radiation exposure) was reported. Imaging studies demonstrated multiple intra-luminal and intra-mural uterine masses with peritoneal deposits. The patient underwent TAH+BSO with excision of peritoneal deposits. The specimen was preserved in 10% formalin, and referred to Pathology Department Lab, Faculty of Medicine, Tanta University, Egypt. Patient's clinical data including name, age, medical and surgical history, contact information & type of operation performed were all recorded.

### Gross examination

The specimen was registered, coded and underwent pathological analysis. Pathological aspects that were assessed included the tumor site, tumor size & extension. Meticulous sampling of the tumor was performed (one section for every 2 cm of the tumor). All submitted sections from the primary uterine tumor obtained from the received specimen were readily available for histopathological examination and further immunohistochemical studies. Formalin-fixed paraffin-embedded (FFPE) tissues were processed for light microscopic examination, and histological sections were stained using hematoxylin and eosin (H&E) stains. Paraffin blocks were then selected for immunohistochemical procedures.

### Histopathological examination

Histopathological features which were evaluated included pattern of growth, presence of any epithelial elements, presence of other heterologous elements, cellular features, nuclear pleomorphism, mitotic activity, amount of rhabdomyoblastic cells, myometrial invasion, vascular invasion and extra-uterine extension.

### Immunohistochemistry

Immunohistochemical studies were performed on FFPE selected blocks from the tumor. The (FFPE) blocks were sectioned (5 µm thick) on positively charged slides and were dried for 30 min at 37°C. The slides were placed in Dako PT Link unit for deparaffinization and antigen retrieval. EnVisionTM FLEX Target Retrieval Solution with a high pH was used at 97°C for 20 minutes. Immunohistochemistry was performed using Dako Autostainer Link 48. For 10 minutes, slides were immersed in Peroxidase-Blocking Reagent, incubated with primary antibodies utilized in this study (summarized in Table 1). Following that, the slides were treated for 20 minutes with horseradish peroxidase polymer reagent and 10 minutes with diaminobenzidine chromogen. After that, the slides were counterstained with hematoxylin.

**Table 1:** Primary antibodies utilized in immunohistochemical study

1ry Antibody	Clone	Source	Dilution
Vimentin	clone V9	Agilent, Santa Clara, United States	FLEX Ready-to-Use primary antibodies
Desmin	clone D33		
Myogenin	clone F5D		
CD56	clone 123C3		
WT-1	clone 6F-H2		
SMA	clone SMMS-1		
CD10	clone 56C6		
ER	Clone EP1		
Cyclin D1	clone EP12		
CD99	clone 12EP		
S100	Polyclonal		
LCA	clone 2B11 + PD7/26		
Pan CK	clone AE1/AE3		
Olig-2	Polyclonal	Abcam, Cambridge, United Kingdom	1:500

### Follow up data

Clinical & follow up information were all obtained from patient medical record and by contacting the referring physician & patient family as well.

### Literature review

A systematic review of the English-language literature since 1972 for “primary uterine rhabdomyosarcoma” in adults above 30 years of age was conducted.

## Results

### Gross examination

**Fig. 1:**
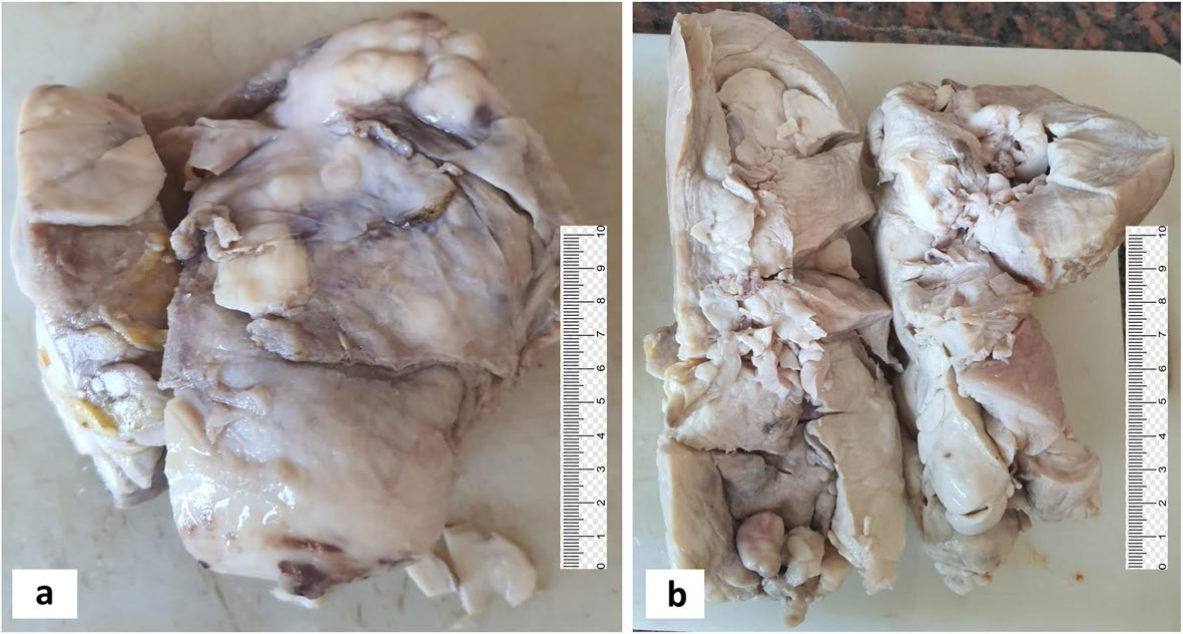
Gross examination of hysterectomy specimen showing white yellowish multinodular solid masses in the uterine corpus encroaching the perimetrium (**a**) & some protruding into the uterine cavity (**b**)

The uterine corpus was cut open when received, measured 18x18x15 cm, and revealed multiple pale spherical firm transmural nodules infiltrating the myometrium and encroaching the perimetrium. Meanwhile, some of these nodules were seen protruding into the uterine cavity. The largest nodule measured 12x7 cm and was centered in the myometrium. All nodules were fleshy, white yellow and homogenous (Figure 1a, b), yet no gross necrosis was seen. The cervical stump was received as a separate specimen measured 9x7x7 cm and showed almost total infiltration by similar nodules. Both ovaries & fallopian tubes were included with each ovary measured about 4x2x1 cm and each tube length was about 7 cm with no remarkable findings. Excised fragmented peritoneal fat measured collectively about 5x3 cm and was studded with metastatic deposits that exhibited similar gross features to the uterine ones.

### Microscopic examination

H&E-stained sections obtained from tumor nodules demonstrated, interestingly, the tumor exhibiting mixed patterns; while the majority of malignant cells were arranged in nests with loss of cellular cohesion in the center giving alveolar pattern, and separated by fibrovascular septa, other areas demonstrating alternating hypo- and hypercellularity within myxoid background with perivascular and sub-epithelial condensation were seen as well. Alveolar areas showed primitive mesenchymal malignant cells with various stages of myogenic differentiation. The tumor cells were mix of medium and large sized, round undifferentiated cells together with differentiating rhabdomyoblastic cells showing eccentric nuclei, frequently with prominent nucleoli, and abundant polygonal eosinophilic cytoplasm with notable cross striations. Other areas were formed of primitive small and medium sized mesenchymal cells that showed lesser degree of striated muscle differentiation with frequent anaplastic cells showing large hyperchromatic nuclei with frequent mitosis. Besides, solid and densely cellular areas showing aggregates of pleomorphic cells with bizarre-looking nuclei and multinucleated tumor giant cells were seen.

**Fig. 2:**
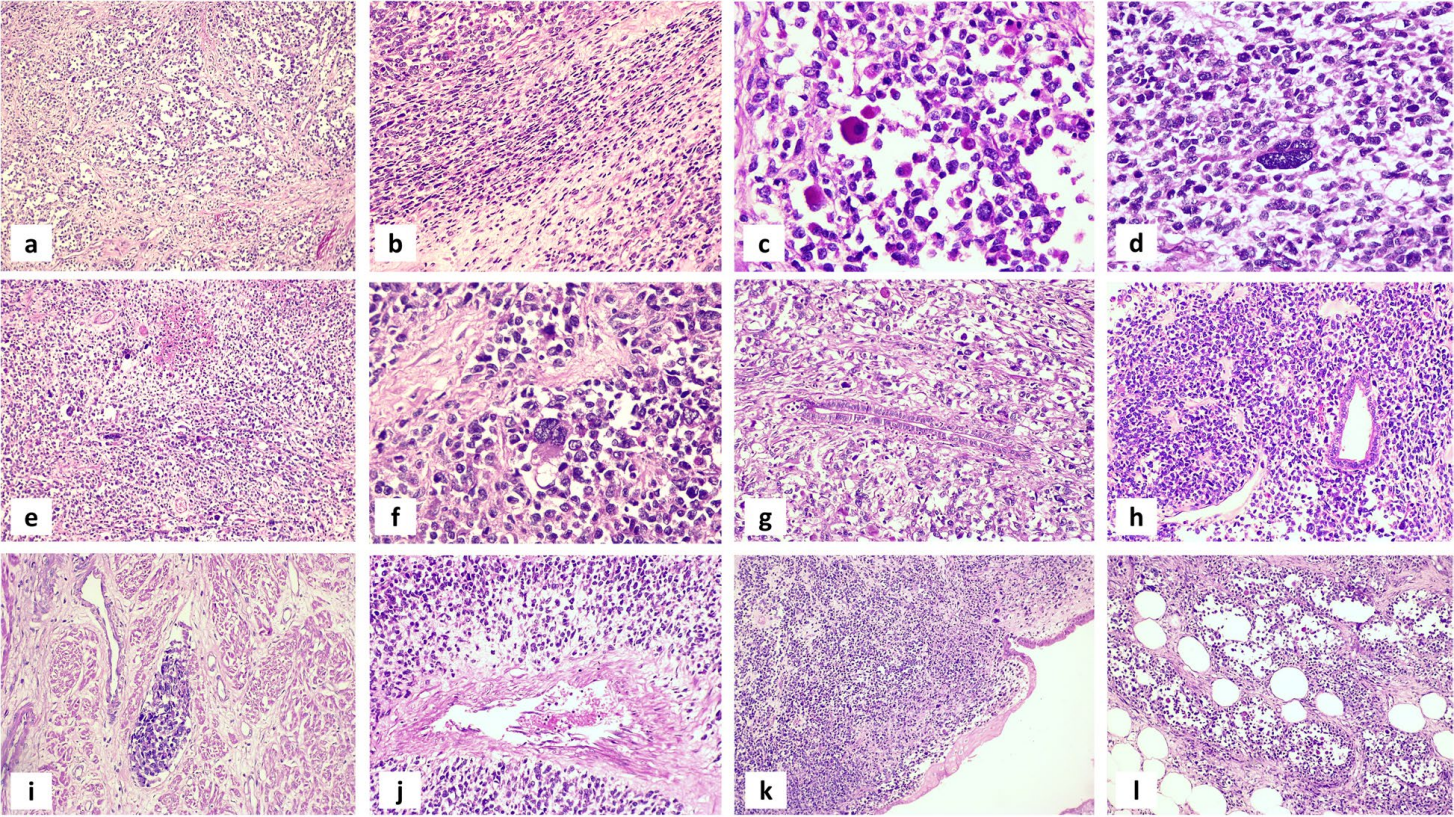
Microscopic examination of studied uterine rhabdomyosarcoma case showing mixed patterns; nests of malignant cells with alveolar pattern (**a**), hypo- and hypercellular areas (**b**) Alveolar areas showing medium and large sized, round undifferentiated cells together with differentiating rhabdomyoblastic cells (**c**), other areas formed of primitive small and medium sized mesenchymal cells frequent anaplastic cells & frequent mitosis (**d**), solid densely cellular areas showing microscopic necrosis, pleomorphic cells with bizarre-looking nuclei and multinucleated tumor giant cells (**e **&**f**). The tumor was diffusely infiltrating uterine wall with entrapment of benign endometrial glands (**g**, **h**), showing lymphovascular emboli (**i**), with perivascular arrangement of tumor cells (**j**) infiltration of cervix (**k**) & peritoneal fat (**l**). [Hematoxylin & Eosin (**a**, **e**, **k**, **l** ) X 100; (**c**, **d** &**f** X 400); (**b**, **g**, **h**, **i**, **j** X 200)]

The tumor was diffusely infiltrating uterine wall (corpus and cervical stump), dissecting the myometrium up to serosa. Although scarce entrapped benign endometrial and endocervical glands were encountered, no malignant epithelial component was detected (the tumor was re-sectioned and thoroughly examined to ensure absence of any neoplastic epithelial element whether adenomatous or carcinomatous). Frequent lymphovascular and perineural invasion was seen together with infiltration of peritoneal fat. Figure 2 (a-l) demonstrates different histopathological features of studied case.

### Immunohistochemistry

**Fig. 3:**
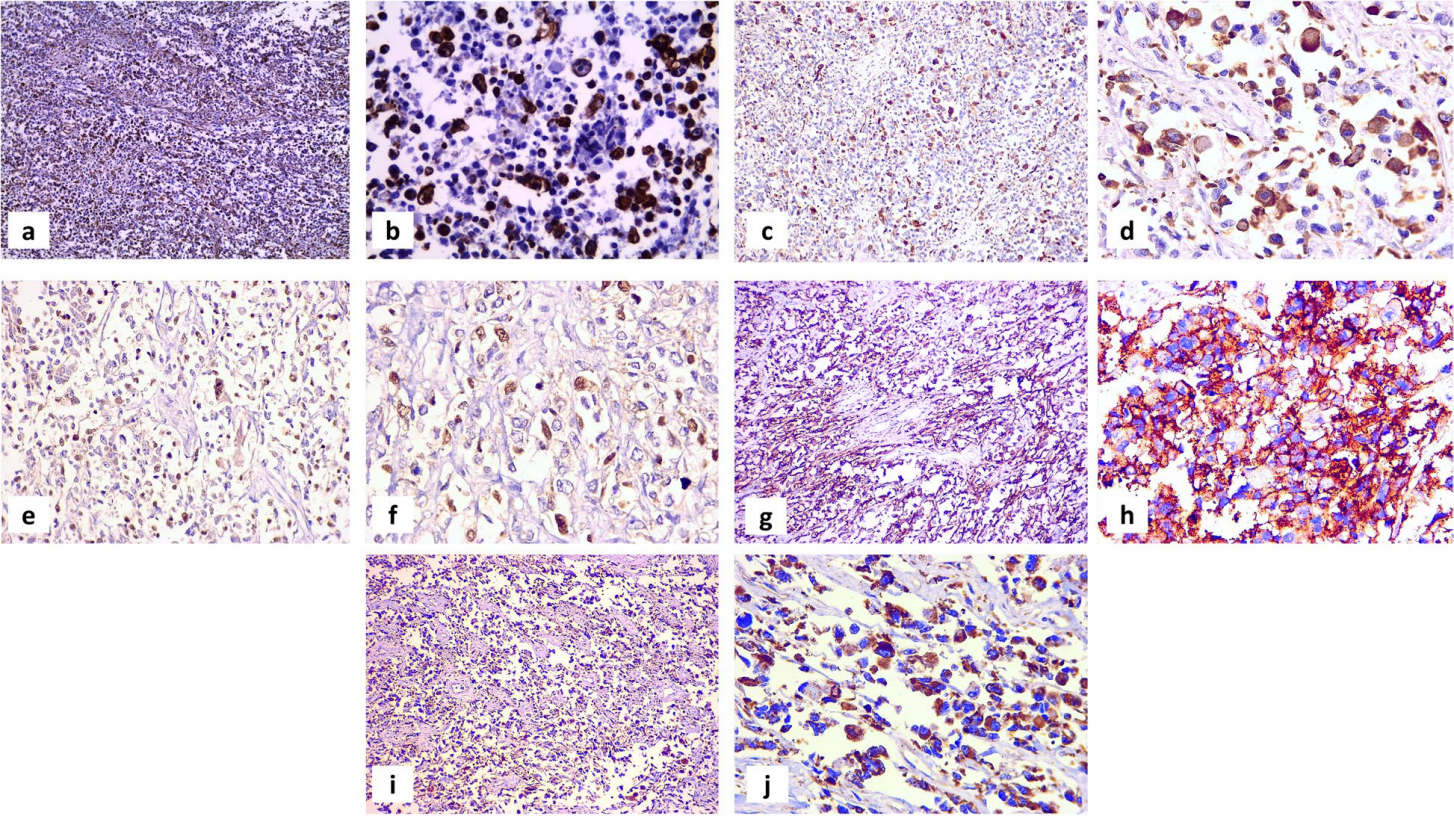
Positive Immunohistochemical DAB staining of tumor cells to Vimentin (a X 100, b X 400); Desmin (c X 100, d X 400) ; Myogenin (e X100, f X 400); CD56 (g X 100, h X 400); WT1 (i X 100, j X 400)

Both vimentin and desmin showed diffuse heterogeneous strong positive cytoplasmic staining (Figure 3: a-d). Also, myogenin showed heterogeneous positive nuclear staining but of moderate-intensity with accentuation in alveolar areas and rhabdomyoblastic cells (Figure 3: e, f). Tumor cells showed membranous positivity for CD56 & cytoplasmic positivity for WT-1 (Figure 3: g-j). SMA, CD10, ER, cyclin D1, CD99, S100, and LCA were all negative. No malignant epithelial element was distinguished with pan cytokeratin or ER. OLIG2 was negative as well.

### Follow up data

The patient received postoperative chemotherapy and radiotherapy but died because of complications of systemic metastases 3 months after surgery.

### Diagnosis and tumour stage

The final diagnosis was primary uterine rhabdomyosarcoma with mixed pattern (embryonal and alveolar). Based on the TNM staging system for uterine sarcoma endorsed by the American Joint Committee on Cancer (AJCC) and the parallel system formulated by the International Federation of Gynecology and Obstetrics (FIGO) 2018 update [11], Tumour stage was pT2NxM1, Stage Group & FIGO Stage IVB. 

### Literature review

The reported cases retrieved by systematic review were summarized and tabulated in a chronological manner (Table 2).

**Table 2:** Reported cases of uterine rhabdomyosarcoma in adults above 30 years of age in literature (1972–2023): [2, 4, 6, 12–48]

References	Age (Ys)	RMS Type	Tumor 1ry Site	Tumor size/ weight	Treatment	Prognosis
Donkers et al. [12] (2 cases)	90	ERMS	Uterine corpus	450 g	Abdominal pan-hysterectomy + excision of parametria	Died 7 months after surgery (?? Sudden heart failure)
	56	PRMS	Uterine corpus	Uterus weighed 150 g	Abdominal pan-hysterectomy + Pre and postoperative CT and RT	DOD 6 1/2 years after surgery
Hart And Craig [13] (3 cases)	42	ُُُ ERMS botryoid	Cervix	5 × 6 cm	TH + BSO + PPLND + post operative RT and adj CT	Alive, no recurrence at 2.7 year F/U after surgery
	70	PRMS	Uterine corpus	14 cm in diameter	TAH + BSO	Death 4 months and 1 week after surgery
	56	PRMS	Uterine corpus (extend to cervix and vagina)	Uterus measured 25 × 16 × 14 cm. and was filled with necrotic tumor,	Palliative CT	Death 6 weeks after diagnosis
Vakiani et al. [14]	68	PRMS	Uterine corpus	7 × 6x5 cm	TH + BSO + post-operative CT	DOD 14 months after diagnosis
Siegal et al. [15]	69	PRMS	Uterine corpus (extend to cervix)	6.1 × 5x4.9 cm	TAH + PPLND + adj RT	Alive, No recurrence at 3 month F/U after surgery
Jaworski et al. [16]	71	PRMS	Uterus	Uterus weighed 1200 g and measured 22x 20 × 70 cm, mass entirely replace uterine corpus	TAH + BSO and patient refused further treatment with CT or RT	Alive, No recurrence at 7 month F/U after surgery
Montag et al. [17]	42	ERMS	Uterine corpus and cervix	6 × 5 cm (largest mass was in the cervix) And separate polypoid mass in endometrium	TH + BSO + PPLND + post-operative RT resection of the upper lobe of the right lung (metastasis), then CT	Alive, no recurrence at 10 year F/U post-operative
Podczaski et al. [18]	73	PRMS	Uterine corpus (extend to cervix	10 × 7 × 5 cm	TAH + BO + Lt S + PPLND + partial O + adj RT	DOD 3 months after surgery
Emerich et al. [19]	45	ARMS	Cervix	10 × 10 × 1.5–2 cm/ 100 g	AH + PLND + adj RT	DOD 3 months after surgery
Chiarle et al. [20]	80	ARMS	Uterine corpus	9 cm in diameter	TAH + BSO + adj RT	DOD 5 months after diagnosis
Ordi et al. [21] (8 cases)	67	PRMS	Cervix	3 cm	TH + adj CT	DOD, 3 weeks after surgery
	87	PRMS	Uterine corpus	1.3 cm	Preoperative RT + TH	Died 1 month after surgery due to pulmonary embolism
	61	PRMS	Uterine corpus (extend to cervix)	8 cm	TH + adj CT and RT	DOD 15 months after surgery
	58	PRMS	Uterine corpus	1.5 cm	Preoperative RT + TH	DOD 12 months after surgery
	69	PRMS	Uterine corpus	5.5 cm (largest)	TH + adj CT and RT	DOD 6 months after surgery
	35	PRMS	Uterus	9.5 cm	TH + adj CT	DOD 15 months after surgery
	80	PRMS	Uterine corpus	13 cm	TH + adj CT	DOD 9 months after surgery
	60	PRMS	Uterine corpus	15 cm	TH + adj CT	Developed breast carcinoma 3 years later and died of a presumed pancreatic carcinoma 6 years later
Holcomb et al. [22]	63	PRMS	Uterine corpus (with endocervical extension)	6 × 6 × 2 cm	TAH + BSO Palliative RT 20 months after surgery	Death 2 years after surgery due to renal failure
Okada et al. [23]	53	PRMS	Uterine corpus (extension to cervix)	Multiple nodules, largest 15 cm	RH + adj RT	Recurrence of disease 2 and 1/2 months after surgery
Takano et al. [24]	76	ERMS	Uterine corpus	15 × 15x 17 cm	TH + BSO + adj CT	Alive, no recurrence at 10 month F/U after surgery
Mccluggage et al. [25]	67	PRMS	Uterine corpus	5 cm polyp + 2 kg of necrotic tumor in pelvis and abdomen	sTH + BSO	Death 3 days after surgery due to renal failure
Ng et al. [26]	39	ARMS	Cervix	6 × 6 cm	TH + Lt SO + adj CT and RT	Alive, no recurrence at 3 year F/U after surgery
Borka et al. [27]	67	PRMS	Uterus	15 cm in greatest dimension	TAH + BSO + PPLND + adj CT	Alive, no recurrence at 12 month F/U after surgery
Reynolds et al. [28]	65	ERMS	Uterine corpus (extends to cervix)	27 × 17 × 15 cm	H with partial cervical excision + BSO + partial cystectomy + O + PLND	DOD 40 days after surgery
Ferguson et al. [29] (9 cases)	56	ERMS botryoid	Cervix	unavailable	TAH + PPLND + adj CT	Alive, No recurrence, at 37 months F/U
	51	ERMS	Cervix	unavailable	TAH + BSO + PPLND + adj CT	Alive, No recurrence, at 7 month F/U
	32	ERMS botryoid	Cervix	unavailable	TAH + BSO + upper vaginectomy + PPLND + adj CT and RT	Alive, no evidence of disease at 125 month F/U
	52	ERMS	Cervix	unavailable	TAH + BSO + PPLND + adj RT	DOD after 17 months
	46	ERMS botryoid	Cervix	unavailable	TAH + BSO + PPLND	DOD after 12 months
	58	ERMS botryoid	Cervix	unavailable	TAH(prior BSO) + PPLND + adj RT	Alive, No recurrence at 27 month F/U
	69	PRMS	Uterine corpus	unavailable	TAH + BSO + adj RT	DOD after 5 months
	45	ERMS botryoid	Uterine corpus	unavailable	TAH + BSO + PPLND + O + adj CT and RT	DOD after 11 months
	49	ERMS botryoid	Uterine corpus	unavailable	RH + PPLND + adj RT	Alive, No recurrence at 11 month F/U
Gottwald et al. [30]	67	Mixed RMS (embryo-nal pleo-morphic, and solid alveolar)	Uterine corpus (extend to cervix)	10 × 6x7 cm	TAH + BSO + PLND + partial lt iliac LN excision + adj CT	Death few weeks after surgery
Rivasi et al. [31]	49	ARMS	Uterine cervix	4 cm	AH (BSO and vaginal biopsy performed after diagnosis) adj CT and RT were refused	Alive, no recurrence at 18 month F/U
Yeasmin et al. [32]	60	PRMS	Uterus	7 × 10x12 cm	TH + BSO + Lt PPLND + Partial O adj CT and RT	DOD 18 months after surgery
Leung et al. [33]	68	PRMS	Uterine corpus	5 × 5 × 2 cm	TAH + BSO	Alive, no recurrence at 12 month F/U after surgery
Chmaj-Wierzchowska et al. [34]	66	PRMS	Uterine corpus	Uterus size: 6 × 7 cm	TH + BSO + adj CT	DOD 2.5 years after surgery
Fadare et al. [35] (4 cases)	51	PRMS	Uterine corpus (1 case extend to the cervix)	Unavailable	TH + BSO + Rt PLND + O	DOD after 0.5 months
	74	PRMS		Unavailable	TH + BSO + PPLND + Orefused adj CT	Presumed recurrence at 6 months, subsequent loss F/U
	79	PRMS		Unavailable	TH + BSO + PPLND + O + adj RT	DOD after 6.3 months
	68	PRMS		Unavailable	TH + BSO + PPLND + O + adj CT and RT	DOD after 19 months
Fukunaga [36]	72	ARMS	Uterine Corpus	6 cm (largest nodule)	TAH + BSO + PLND + tumorectomy of a retroperitoneal mass + adj CT	DOD 12 months after surgery
Kriseman et al. [37] (4 cases)	33.3	Not defined for each patient ERMS: botryoid and non botryoid & undiffer-entiated RMS	Cervix	Unavailable	TAH + BSO + adj CT and RT	Death of CT complication
	49.3		Cervix and lower uterine segment	Unavailable	TAH + BSO + adj CT and RT	Death of parotid adenocarcinoma
	51.9		Cervix	Unavailable	Cone biopsy(no available data about adj treatment)	Alive, at 19 months F/U
	34.2		Cervix	Unavailable	CT + adj cone biopsy	Death of unknown cause
Kim et al. [38]	76	ERMS Spindle cell	Uterine corpus	20 × 15 × 7 cm	sTH + BSO Refused further treatment	DOD 3 months after diagnosis
Li et al. [39] ( 18 cases)	56	ERMS	Cervix	Unavailable	Unavailable	Unavailable
	51	ERMS	Cervix	Unavailable	Unavailable	Unavailable
	47	ERMS	Cervix	Unavailable	Unavailable	Unavailable
	31	ERMS	Cervix	Unavailable	Unavailable	Unavailable
	44	ERMS	Cervix	Unavailable	Unavailable	Unavailable
	46	ERMS	Cervix	Unavailable	Unavailable	Unavailable
	48	ERMS	Cervix	Unavailable	Unavailable	Unavailable
	49	ERMS	Cervix	Unavailable	SH	Unavailable
	46	ERMS	Cervix	Unavailable	RH + adj CT	Alive, no recurrence at 3 year F/U
	56	ERMS	Cervix	Unavailable	Unavailable	Unavailable
	73	ERMS	Cervix	Unavailable	No treatment was received	DOD after 5 months (pulmonary metastasis)
	43	ERMS	Cervix	Unavailable	RH	Unavailable
	54	ERMS	Cervix	Unavailable	RH, elected for CT	AWD at 5 month F/U
	89	ERMS	Cervix	Unavailable	Unavailable	Unavailable
	52	ERMS	Uterine corpus	Unavailable	Hysterectomy (unspecified)	Alive, no recurrence at 3 year F/U
	43	ERMS	Uterine corpus	Unavailable	Unavailable	Unavailable
	48	ERMS	Uterine corpus	Unavailable	Unavailable	Unavailable
	63	ERMS	Uterine corpus	Unavailable	Unavailable	Unavailable
Kuroki et al. [40]	36	Not defined	Uterus	Unavailable	CT	Death 5 months after diagnosis
Yamada et al. [41]	55	ERMS	Uterine corpus	11 × 7 cm	TAH + BSO	Alive, no recurrence at 6 month F/U
Alavi et al. [42]	73	PRMS	Uterine corpus (with invasion of cervix)	6.5 × 6 × 5 cm	AH + BSO (elected for RT but treatment was rejected by patient)	Unavailable
Pinto et al. [4] (7 cases) The type of surgical procedures for each case was unavailable	40	ARMS	Uterine corpus with cervical extension	12 cm	CT	AWD at 18 month F/U
	68	PRMS	Uterine corpus	13.6 cm	CT	DOD after 10 months
	65	PRMS	Uterine corpus	Unavailable	CT	AWD at 26 month F/U
	62	PRMS	Uterine corpus withcervical extension	15.2 cm	Hospice care(patient refused CT)	DOD after 4 months
	70	PRMS	Uterine corpus with cervical extension	13 cm	CT	AWD at 9 month F/U
	64	ARMS	Uterine corpus	14.5 cm	Hospice care(patient refused CT)	DOD after 6 weeks
	48	ERMS	Cervix	6.0 cm	Unavailable	Unavailable
Motoda et al. [43]	50	ARMS	Uterus	10 cm	Partial resection	Death 19 days after surgery
Aljehani et al. [44]	54	ERMS	Uterus	10 cm in aggregates	Palliative RT	Unavailable
Amini-moghaddam et al. [45]	60	ARMS	Uterine corpus	10 × 8 × 6 cm	TH + BSO + PPLND + neoadj CT + adj RT	Alive, no recurrence at 12 month F/U
Choi et al. [46]	90	ARMS	Uterus	19 cm	TAH + BSO + PPLND	Death at 8th day post-operative
Li et al. [6]	81	PRMS	Uterine corpus	Uterus size/ weight: 26 cm /5.2 kg	TAH + BSO + O (adj CT or RT refused by the patient)	Alive, No recurrence at 4 month F/U after surgery
Nishino et al. [2]	51	ARMS	Uterine corpus (extends to cervix)	Uterus size: 11.5 × 9.5 × 4 cm	TH + BSO + adj CT	AWD at 6 month F/U
Tamura et al. [47]	58	PRMS	Uterus	Unavailable	RH	Unavailable
Kamboj et al. [48]	53	PRMS	Uterine corpus	11.5 × 11.5 × 9 cm	RH + adj CT	Unavailable

*ARMS *Alveolar rhabdomyosarcoma, *ERMS *Embryonal rhabdomyosarcoma, *PRMS *Pleomorphic rhabdomyosarcoma, *TAH *Total abdominal hysterectomy, *TH *Total hysterectomy, *sTH *Subtotal hysterectomy, *SH *Simple hysterectomy, *AH *Abdominal hysterectomy, *RH *Radical hysterectomy, *PPLND *Pelvic and paraaortic L.N dissection, *PLND *Pelvic L.N dissection, *Lt PPLND *Left pelvic and paraaortic L.N dissection, *Rt PLND *Right pelvic L.N dissection, *O *Omentectomy, *adj *adjuvant, *neoadj *neoadjuvant, *RT *Radiotherapy, *CT *Chemotherapy, *BSO *Bilateral salpingo-oophorectomy, *BO *Bilateral oophorectomy, *Lt SO *Left salpingo-oophorectomy, *Lt S *Left salpingectomy, *DOD *Death of disease, *AWD *Alive with disease, *F/U *Follow up

## Discussion

The current study handled a very rare and interesting case of a primary uterine mixed embryonal and alveolar type rhabdomyosarcoma involving both uterine corpus and cervix in a 68-year old woman, which provided an opportunity to enlighten different aspects regarding the diagnosis and differential diagnosis of primary uterine RMS as well as better understanding of RMS classification and characteristics of each subtype by surveying recent related publications. 

The systematic review of the English-language literature that focused on primary uterine rhabdomyosarcoma in adults above 30 years of age uncovered 87 cases between 1972 and 2023. Recorded available variables, including age, RMS type, tumor size/weight, treatment methods, and follow-up are shown in Table 2. To our knowledge this is the broadest literature review collection of such rare cases.

Mixed pattern RMS (ARMS and ERMS) constitutes a diagnostic dilemma regarding its histopathological features. Whereas some confusion may easily occur between ARMS cases that show solid areas reminiscent of ERMS and ERMS cases with dense pattern that may resemble solid ARMS, the truly histologically mixed pattern rhabdomyosarcomas are rare tumors and applied only for selected cases. These tumors exhibit separate, discrete ARMS and ERMS morphology with variable extent of each component [49]. Originally, it was sufficient to establish the diagnosis of ARMS if any focus of alveolar morphology was identified, and tumors that exhibit discrete areas of both alveolar and embryonal histology "of any histologic pattern of ERMS” were diagnosed as ARMS [50, 51].

In cases of malignant mesenchymal tumor in the uterus, extensive sampling is necessary to exclude sarcomatous overgrowth in adenosarcoma or carcinosarcoma[51, 52]. Adenosarcoma is generally characterized by broad leaf-like or club-like projections[53]. In the present case, extensive sampling of surgical specimen and cytokeratin immunostaining failed to reveal the presence of any neoplastic epithelial elements, leading to the adenosarcoma and carcinosarcoma diagnoses being ruled out.

The tumor cells were immunohistochemically positive for vimentin, also they were positive for striated muscle markers, such as desmin & myogenin but negative for SMA. These findings were similar to those reported by others [39]. Expressions of both desmin & myogenin are reciprocally related to the degree of cellular differentiation, thus more myogenin staining is seen in primitive-appearing cells and a decreased or absence of immunoreactivity is seen in large differentiated rhabdomyoblasts and the opposite reported for desmin [54].

Endometrial stromal sarcoma was excluded in this case by negative immunostaining to CD10, ER, CD99, and Cyclin-D1 primary antibodies. WT-1 showed only cytoplasmic staining with absent nuclear staining, supporting the idea that tumors with this phenotype exhibit WT1 deregulation. The immunohistochemical results were in line with previous findings that WT-1 protein is not acting as a nuclear transcription factor in such tumors but instead is stabilized in the cytoplasm [55].

CD56 showed membranous staining in tumor cells, which is a sensitive marker of poorly differentiated neuroendocrine carcinomas. However, the results highlight the lack of specificity of this antibody, especially in clinical situations where small cell carcinoma is suspected. Moreover, Bahrami et al., reported in 2008 that it may also be expressed in almost all other small round cell neoplasms [56]. Results of CD56 expression in current case are in keeping with these prior findings.

One of the important implications of findings in presented case was recognition that ARMS can display a wide immunophenotypical spectrum, and this grabbed attention to avoid misdiagnosis of this tumor as it morphologically can resembles other small round cell tumors.

The histogenesis of rhabdomyosarcomatous differentiation in uterine RMS is not fully understood, but it could arise from primitive or uncommitted mesenchymal cells that undergo rhabdomyosarcomatous differentiation. An alternative theory suggests that uterine RMS represents sarcomatous overgrowth in adenosarcoma or carcinosarcoma, although this would be difficult to prove in practice [57].

The chromosomal translocations t(2;13)(q35;q14) and t(1;13)(p36;q14) are characteristic of soft tissue alveolar rhabdomyosarcoma. Molecular classification has been proposed, dividing RMS into two basic groups: fusion-positive RMS (either PAX7::FOXO1 enriched or PAX3::FOXO1 enriched) and fusion negative RMS (which is further sub-divided into well differentiated RMS, moderately differentiated RMS, and undifferentiated sarcomas) [58]. ERMS and PRMS are typically fusion negative. Whereas ARMS with t(2;13) & PAX3::FOXO1 translocations has a worse prognosis compared to PAX7::FOXO1 and fusion negative cases of ARMS [59]. Recent publications reported that the remaining fraction of fusion-negative ARMS have a clinical and biological behavior similar to ERMS[60].

The fusion status of RMS with mixed patterns is heterogeneous among different publications, but the majority of reported cases are fusion-negative [58]. It is believed that fusion status for all cases of RMS, including RMS with mixed-pattern, should be investigated since it carries a prognostic value. Several studies have examined gene expression differences in fusion-driven RMS compared to its fusion-negative counterpart,as well as their relation to myogenin expression status, and reported that strong and diffuse expression of myogenin is closely associated with the presence of PAX3/7::FOXO1 translocations [61–63]. Kaleta et al., in 2019 concluded that immunohistochemical expression of OLIG2 may function as a surrogate marker for the presence of PAX3/7::FOXO1 translocation in RMS [64]. The current case showed no evidence of OLIG2 immunohistochemical staining and heterogeneous expression of myogenin, possibly denoting fusion negativity. One of the shortages of this study is that genetic analysis was not performed, and thus we emphasize on the importance of molecular testing for accurate categorization and better predilection of the tumor behavior.

Rhabdomyosarcoma arising in the uterus has been fairly reported. In 1909, Robertsondescribed the first case of uterine rhabdomyosarcoma in English literature, where an alveolar architecture for the tumor was portrayed [65]. Nevertheless, mixed rhabdomyosarcoma of the alveolar and embryonal types is very rare. To the best of our knowledge, besides the present case, only Gottwald et al., in 2008, reported such case. They reported that she had previous history of breast carcinoma, and interestingly, was diagnosed with both uterine RMS and Gastric GIST while receiving adjuvant hormonal therapy for breast cancer [30]. The present case had no past medical history, yet pursued a very aggressive clinical course and died 3 months after surgery because of complications of systemic metastasis,despite receiving postoperative chemotherapy and radiotherapy.

## Conclusion

Summing up, the above-described clinical case of rhabdomyosarcoma with mixed alveolar & embryonal patterns of adult uterus is a very rare malignant tumor. Its diagnosis is based on histopathological analysis and confirmed by immunohistochemical examination. Clinical symptoms are non-specific for these cases. The rarity of this histological entity and protocol applied make the presented case worthy to shed light on. Moreover, despite comprehensive treatment, it is an aggressive tumor with poor prognosis and thus further molecular studies & research are needed to improve therapy options in adults.

### Ethical statement

Approval for a study protocol was not required because this was a case report with literature review. The authors have obtained the patient’s written informed consent for print and electronic publication of this case report.

## Data Availability

No datasets were generated or analysed during the current study.

## Abbreviations

RMS: Rhabdomyosarcoma
ERMS: Embryonal rhabdomyosarcoma
ARMS: Alveolar rhabdomyosarcoma
PRMS: Pleomorphic rhabdomyosarcoma
LMS: Leiomyosarcoma
ESS: Endometrial stromal sarcoma
WHO: World Health Organization
TNM: Tumor (T), nodes (N), and metastases (M)
AJCC: American Joint Committee on Cancer
FIGO: The International Federation of Gynecology and Obstetrics
FFPE: Formalin-fixed paraffin-embedded
H and E: Heamatoxylin and eosin
TAH: Total abdominal hysterectomy
SH: Simple hysterectomy
sTH: Subtotal hysterectomy
TH: Total hysterectomy
AH: Abdominal hysterectomy
RH: Radical hysterectomy
Lt S: Left salpingectomy
PPLND: Pelvic and paraaortic L.N dissection
PLND: Pelvic L.N dissection
Lt PPLND: Left pelvic and paraaortic L.N dissection
Rt PLND: Right pelvic L.N dissection
O: Omentectomy
adj: Adjuvant
neoadj: Neoadjuvant
RT: Radiotherapy
CT: Chemotherapy
BSO: Bilateral salpingo-oophorectomy
BO: Bilateral oophorectomy
Lt SO: Left salpingo-oophorectomy
DOD: Death of disease
AWD: Alive with disease
F/U: Follow up
